# 

**Published:** 1884-03

**Authors:** 


### CONTENTS.

COMMUNICATIONS.

On Certain Microscopic Elements in Pulpless and Gum-Denuded Teeth, in their Relations to the Filling of Roots, and the Re-Attachment of the Gum-Tissue........ 111
What is Meant by Nervous Prostration........ 126

SELECTIONS.

Rules of Business........ 136
A Simple Method of Producing a Reticulated Surface on the Palatal Side of Dental Bas-Plates........ 139
Acid and Fungus........ 139
The Relation of Physiological Study to Pathology and Therapeutics. 140
Storage-Battery Light........ 142
Corrosive Sublimate as an Antiseptic........ 142
Horse-Back Riding and Pulmonary Calisthenics........ 143
White Fillings........ 143
The Folly of Anti-Vaccinators........ 144
Iodoform Intoxication........ 146
What to do with our Sons........ 147
Trees in Towns........ 147
The Causes and Prevention of Suicide........ 148
Micro-Organisms in Water........ 148
Malaria Night-Blindness........ 148
The Action of Saline Cathartics........ 150
The Use of Tobacco by Boys........ 150
Odonto-Periosteal Tumor Simulating Pulp—Fungoid........ 152
Correspondence........ 154
Salmagundi........ 154

EDITORIAL.

A New Combination........ 160
The Alabama State Dental Association........ 160
Michigan Dental Society........ 161
Book Notices........ 161

